# EXERCISE CAPACITY IN CHILDREN AND ADOLESCENTS WITH POST-INFECTIOUS BRONCHIOLITIS OBLITERANS: A SYSTEMATIC REVIEW

**DOI:** 10.1590/1984-0462/;2019;37;2;00017

**Published:** 2019-03-18

**Authors:** Camila Menna Barros Rodrigues, Daniele Schiwe, Natália Evangelista de Campos, Fabiana Niederauer, João Paulo Heinzmann-Filho

**Affiliations:** a Hospital Moinhos de Vento, Porto Alegre, RS, Brazil.; b Pontifícia Universidade Católica do Rio Grande do Sul, Porto Alegre, RS, Brazil.

**Keywords:** Exercise capacity, Physical fitness, Physical activity, Bronchiolitis obliterans, Pediatrics, Capacidade de exercício, Aptidão física, Atividade física, Bronquiolite Obliterante, Pediatria

## Abstract

**Objective:**

To evaluate exercise capacity in children and adolescents with post-infectious bronchiolitis obliterans.

**Data source:**

This is a systematic review based on data from PubMed, Literatura Latino-Americana e do Caribe em Ciências da Saúde (LILACS), Scientific Electronic Library Online (SciELO), and Physiotherapy Evidence Database (PEDro). We used the following search strategy: “Exercise capacity OR Exercise Test OR Physical fitness OR Functional capacity OR Six-minute walk test OR Shuttle walk test OR Cardiopulmonary exercise test AND Bronchiolitis obliterans.” We selected studies that evaluated exercise capacity through maximal/submaximal testing in children and adolescents with post-infectious bronchiolitis obliterans, and no other associated disease. We searched articles in English, Portuguese, and Spanish, without restrictions regarding the period of publication. The methodological quality was assessed by the Agency for Healthcare Research and Quality (AHRQ) protocol.

**Data synthesis:**

Out of the 81 articles found, only 4 were included in this review. The studies totaled 135 participants (121 with post-infectious bronchiolitis obliterans and 14 healthy), with sample sizes between 14 and 58 subjects. All patients underwent spirometry to evaluate pulmonary function, indicating an obstructive ventilatory pattern. Among them, 3/4 had their physical performance assessed by the six-minute walk test and 2/4 by the cardiopulmonary exercise testing. These test results were compared to those of a control group (1/4) and presented as percentage of predicted and/or in meters (3/4). Lastly, 3/4 of the studies showed reduced exercise capacity in this population. The studies included were classified as having high methodological quality.

**Conclusions:**

Findings of the study demonstrate that children and adolescents with post-infectious bronchiolitis obliterans have reduced exercise capacity.

## RESUMO

**Objetivo:**

Avaliar a capacidade de exercício em crianças e adolescentes com bronquiolite obliterante pós-infeciosa.

**Fonte de dados:**

Trata-se de uma revisão sistemática por meio das bases de dados PubMed, Literatura Latino-Americana e do Caribe em Ciências da Saúde (LILACS), Scientific Electronic Library Online (SciELO) e Physiotherapy Evidence Database (PEDro). Utilizou-se a seguinte estratégia de busca: *“Exercise capacity OR Exercise Test OR Physical fitness OR Functional capacity OR Six minute walk test OR Shuttle walk test OR Cardiopulmonary exercise test AND Bronchiolitis obliterans”*. Foram selecionados estudos que avaliaram a capacidade de exercício por meio de testes máximos/submáximos em crianças e adolescentes com bronquiolite obliterante pós-infeciosa, sem qualquer outra doença associada. Buscaram-se artigos nos idiomas inglês, português e espanhol e sem restrições quanto ao período de publicação. A qualidade metodológica foi avaliada pelo protocolo da *Agency for Health Care Research and Quality* (AHRQ).

**Síntese dos dados:**

De um total de 81 artigos, apenas 4 foram incluídos nesta revisão. Os estudos totalizaram 135 participantes (121 com bronquiolite obliterante pós-infeciosa e 14 saudáveis), com tamanho amostral entre 14 e 58 sujeitos. Todos avaliaram a função pulmonar por meio da espirometria, observando um padrão ventilatório obstrutivo. Desses, 3/4 avaliaram o desempenho físico pelo teste de caminhada de seis minutos e 2/4 pelo teste de exercício cardiopulmonar. Os resultados desses testes foram comparados a um grupo controle (1/4), bem como apresentados em percentual do previsto e/ou em metros (3/4). Por fim, 3/4 dos estudos demostraram que a capacidade de exercício se encontra reduzida nessa população. Os estudos incluídos foram classificados com alta qualidade metodológica.

**Conclusões:**

Os achados do estudo demonstram que crianças e adolescentes com bronquiolite obliterante pós-infeciosa apresentam redução da capacidade de exercício.

## INTRODUCTION

Post-infectious bronchiolitis obliterans (PIBO) is an inflammatory disease of the distal airways, resulting from damage to the lower respiratory tract. Inflammation and fibrosis of terminal bronchioles causes narrowing and/or complete obliteration of the airway lumen, leading to chronic airflow obstruction.^1^ ^,^ ^2^ ^,^ ^3^ ^,^ ^4^ ^,^ ^5^ ^,^ ^6^ ^,^ ^7^ These anatomofunctional changes can result in progressive loss of strength/endurance of ventilatory muscles, directly contributing to the reduction in activities of daily living (ADLs), with a consequent negative impact on exercise tolerance.^8^

Reduction in exercise tolerance for individuals with respiratory diseases has a significant association with quality of life, hospitalizations, medicines, survival time, and clinical prognosis.^8^ ^,^ ^9^ ^,^ ^10^ In this regard, field/laboratory functional tests are considered essential components in the routine of clinical evaluation of patients, due to their clear and objective way of measuring functional capacity and the reasons for exercise intolerance, and for prescription of an appropriate physical training program.^11^ ^,^ ^12^ Among these tests, we can mention the cardiopulmonary exercise testing (CPET) and the six-minute walk test (6MWT).^13^ ^,^ ^14^

Current data suggest that results of exercise testings could be more sensitive in detecting early involvement in respiratory diseases when compared to the forced expiratory volume in one second (FEV_1_), measured by spirometry. This fact is due to physical performance tests evaluating the communication of ventilatory, cardiac, and metabolic systems in an integrated and dynamic way, while spirometry is just a static pulmonary measurement.^3^ ^,^ ^15^ ^,^ ^16^ ^,^ ^17^ ^,^ ^18^

Although CPET and 6MWT results are widely known in some chronic lung diseases in children, including cystic fibrosis and asthma,^19^ ^,^ ^20^ ^,^ ^21^ these findings are more restricted in PIBO.^22^ ^,^ ^23^ To date, few studies have evaluated functional capacity in patients with PIBO, especially in the pediatric population.^11^ ^,^ ^24^ In addition, we found no systematic and critical review aimed at evaluating exercise capacity with different functional tests in children and adolescents with PIBO, justifying the development of this study. A better understanding and knowledge about the topic can alert the professionals involved in the care of these patients to the need of designing effective strategic measures to fight exercise intolerance.

## METHOD

This is a systematic review conducted by searching databases from PubMed, Literatura Latino-Americana e do Caribe em Ciências da Saúde (LILACS), Scientific Electronic Library Online (SciELO), and Physiotherapy Evidence Database (PEDro). We selected observational studies in English, Portuguese, and Spanish, without filters regarding age and year of publication of articles. The selection of studies occurred in June 2017.

The search adopted to choose the articles was based on eight keywords associated with Boolean operators. We used the following strategy: “Exercise capacity OR Exercise Test OR Physical fitness OR Functional capacity OR Six-minute walk test OR Shuttle walk test OR Cardiopulmonary exercise test AND Bronchiolitis obliterans”. These terms should appear, at least, in the heading, abstract, or keywords.

We included studies that evaluated exercise capacity using maximal and/or submaximal testing in children and adolescents with PIBO and no other associated disease. Exercise capacity was considered preserved when the results of the distance covered or maximal oxygen uptake were ≥80% of predicted, or when data were compared to a control group. Also, in the absence of previous criteria, the subjects were classified as having preserved physical capacity when they covered a distance ≥476 m on 6MWT.^21^ In contrast, we excluded review studies, case reports, articles that did not assess exercise capacity, and those that evaluated only adults. We also excluded studies involving individuals with other chronic diseases, neurological disorders, orthopedic problems, and/or cognitive limitations.

Later, we identified the terms in the headings, and read the abstracts of the selected articles to assess whether they fit the eligibility criteria. Studies that presented the predetermined criteria had the full text purchased for data extraction and analysis. Two raters searched and analyzed the articles independently, and a third resolved disagreements consensually.

We registered the following characteristics of the studies: name of the first author/year of publication, country (origin) of data collection, groups assessed (PIBO and healthy), age group, sample size, data on pulmonary function (spirometry), exercise testing performed, and main results found (reduced, preserved, or improved physical capacity).

We used a scale suitable for observational studies from the Agency for Health Care Research and Quality (AHRQ).^25^ This instrument evaluates nine items related to the study question, methodological aspects, consistency of results, discussion, and sponsorship. The final sum of each item evaluated is 100 points, with studies classified as having low (<50 points), moderate (50-66 points), and high (>66 points) methodological quality.

## RESULTS

**Figure 1:**
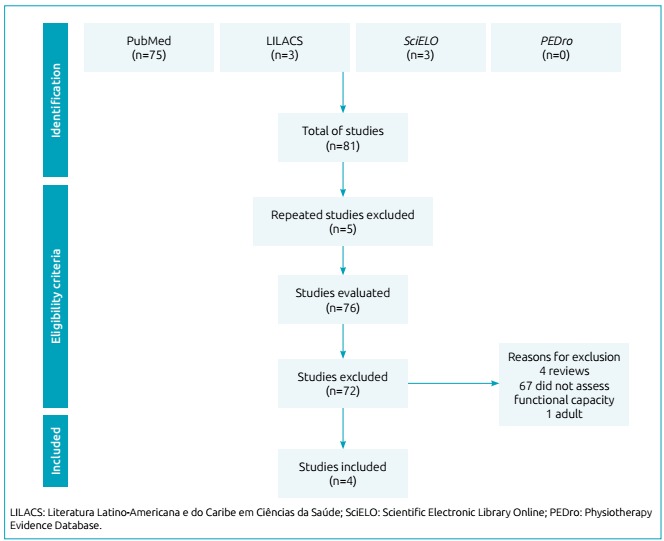
Systematization of the studies included in this systematic review.

Out of 81 articles, 75 were found in PubMed, three in LILACS, three in SciELO, and none in PEDro. We excluded five studies for appearing in more than one of the databases used, and 72 for not meeting the eligibility criteria of this review. Thus, we included only four studies that evaluated exercise capacity in children and adolescents with PIBO. Figure 1 shows the flowchart related to the total number of articles found.

The selected articles totaled a sample of 135 participants (121 with PIBO and 14 healthy). The sample size of the studies varied between 14 and 58 subjects, with ages ranging from 8 to 23 years (Table 1). Two studies (50.0%) evaluated exercise capacity with 6MWT; one (25.0%) with CPET; and one (25.0%) with both tests. Only one study of this review (25.0%) used a control group.

**Table 1:** Identification and characteristics of the studies included in this review.

First author & year	Country (origin)	Groups assessed	Age group (years)	Sample size
Frohlich et al. (2014)^22^	Brazil	Healthy PIBO	10-23 10-23	14 16
Zenteno et al. (2009)^23^	Chile	PIBO	8-14	27
Mattiello et al. (2008)^11^	Brazil	PIBO	8-16	20
Gerbase et al. (2008)^24^	Switzerland	PIBO	5.6±2.9*	58

PIBO: post-infectious bronchiolitis obliterans; *data presented in mean and standard deviation.

Data on pulmonary function (% of predicted) included FEV_1_, forced vital capacity (FVC), the Tiffeneau index (FEV_1_/FVC), and forced expiratory flow in 25 and 75% of FVC (FEF_25-75_), indicating moderate/severe pulmonary involvement. The parameters evaluated with exercise testing included the distance covered (meters and % of predicted) and maximal oxygen uptake (VO_2max._) (% of predicted). Approximately 75.0% of the studies demonstrated that children and adolescents with PIBO had reduced exercise capacity. Table 2 presents this information in detail.

**Table 2:** Characteristics and main results of the studies included in this review.

First author & year	Groups assessed	Pulmonary function (% of predicted)	Exercise testing used	Results of the exercise testing (% of predicted)	Conclusion of the exercise capacity
Frohlich et al. (2014)^22^	Healthy	FEV_1_: 112±15 FVC: 112±16 FEV_1_/FVC: 99±03	CPET	VO_2max._: 101±17	Reduced exercise capacity compared to healthy individuals (p-value<0.05)
	PIBO	FEV_1_: 60±30 FVC: 74±22 FEV_1_/FVC: 78±21		VO_2max._: 84±15	
Zenteno et al. (2009)^23^	PIBO	FVC: 85.4±15 FEV_1_: 58.3±23 FEV_1_/FVC: 59.3±32 FEF_25-75_: 31.7±25	6MWT	Distance covered: 598±71^#^	Preserved exercise capacity (>476 m)
Mattiello et al. (2008)^11^	PIBO	FEV_1_: 57.7±17.9 FVC: 66.8±17.3 FEV_1_/FVC: 57.9±12.5 FEF_25-75_: 20.4±12.6	6MWT CPET	Distance covered: 77±15.7 VO_2max._: 77.5±37.5	Reduced exercise capacity (<80% of predicted)
Gerbase et al. (2008)^24^	PIBO	FEV_1_: 73.9±25.1	6MWT	Distance covered: 30.4 (4.5-8.1)*	Reduced exercise capacity (<80% of predicted)

%: percentage; PIBO: post-infectious bronchiolitis obliterans; FEV_1_: forced expiratory volume in 1 second; FVC: forced vital capacity; FEV_1_/FVC: Tiffeneau index; FEF_25-75_: forced expiratory flow; CPET: cardiopulmonary exercise testing; 6MWT: six-minute walk test; VO_2max._: maximal oxygen uptake; *data expressed in median and variation; ^#^data presented in meters.

The mean score of methodological quality of the selected articles was 77.25 points, ranging from 73 to 82 (Table 3). All studies (100%) reached a score compatible with high methodological quality, according to the AHRQ scale. Factors that decreased the quality score concerned some items related to the population evaluated, comparability of studies (inclusion/exclusion criteria), result measurements (blinding), and statistical analysis (sample calculation).

**Table 3:** Assessment of the methodological quality of the studies included in this systematic review.

Criteria evaluated*	Reference score	Frohlich et al. (2014)^22^	Zenteno et al. (2009)^23^	Mattiello et al. (2008)^11^	Gerbase et al. (2008)^24^
Study question	2	2	2	2	2
Study population	8	5	5	8	5
Comparability of individuals for observational studies	22	19	19	19	17
Exposure or intervention	11	11	11	11	11
Result measurements	20	15	15	15	15
Statistical analysis	19	12	12	14	12
Results	8	8	8	8	8
Discussion	5	5	5	5	3
Funding and sponsorship	5	0	0	0	0
Total score	100	77	77	82	73

*Some items assessed in this scale did not directly apply to the design of the studies.

## DISCUSSION

In this review, we identified four studies^11^ ^,^ ^22^ ^,^ ^23^ ^,^ ^24^ with high methodological quality that evaluated exercise capacity in children and adolescents with PIBO. Among them, 75.0% showed reduced functional capacity, measured both by laboratory (maximal) and field (submaximal) tests. In patients with chronic lung disease, the measurement of physical fitness is considered part of the multidimensional assessment, since it objectively evaluates the interaction among the cardiac, ventilatory, muscular, and metabolic systems.^26^

The main reasons for exercise intolerance in patients with lung disease include isolated or associated factors, such as the enhancement of symptoms (fatigue in lower limbs and dyspnea), development of dynamic hyperinflation, peripheral muscle dysfunction, abnormalities in oxygen transport, and progressive physical deconditioning related to inactivity.^8^ ^,^ ^12^ Furthermore, the lack of ventilatory reserve observed by the high ventilation reached in CPET (almost all of the maximal voluntary ventilation predicted) also seems to be related to the low physical performance of these patients, a fact attributed to the obstructive ventilatory pattern in these subjects, which directly limits the airflow.^17^

In the present review, three studies used 6MWT (two exclusively and one with CPET) to measure exercise capacity in patients with PIBO.^11^ ^,^ ^23^ ^,^ ^24^ Among them, two showed reduced functional capacity,^11^ ^,^ ^24^ while the other^23^ presented values within the cohort point (>476 m), considered the lower normal limit.^21^ Clinically, 6MWT relates to ADLs and investigates the need for supplemental oxygen therapy,^27^ in addition to being more indicated for patients with lung diseases in more advanced stages, due to its low sensitivity in early stages.^27^ ^,^ ^28^ This fact can be observed in this review, considering that 6MWT detected reduced exercise capacity in subjects with moderate/severe pulmonary involvement.

Only the study by Zenteno et al.^23^ was classified as having preserved physical capacity, based on our methodological criteria. However, this interpretation might have been affected by the lack of standardization of results of distance covered by a reference equation. Nonetheless, we used cohort points described in the literature to aid in this categorization.^21^ This cohort point was chosen for being an intermediate value (between 400 and 577.5 m) of the distance covered in 6MWT, used in studies that evaluated best/worst clinical prognosis in patients with lung diseases.^9^ ^,^ ^21^ ^,^ ^28^ Based on the results of this review, 6MWT can be the first choice to assess physical performance in pediatric patients with PIBO who present moderate/severe pulmonary involvement, considering its good detection power for impaired physical performance in these samples.

All studies that assessed physical fitness with CPET (2/4 of the articles: 50 subjects out of 135 - PIBO and control group - and 36 out of 121 - only PIBO) demonstrated exercise intolerance in patients with PIBO. CPET is considered the gold-standard method to investigate physical fitness, obtaining as clinical variables VO_2max._; maximal ventilation; respiratory equivalent for both oxygen and carbon dioxide; and ventilatory and cardiac reserves.^29^ In chronic respiratory diseases, the main outcome used is VO_2max._, a variable adopted in our review to interpret data.^16^ ^,^ ^29^ VO_2max._ has a good relationship with other clinical markers and is often used as an indicator of life expectancy in many lung diseases. Among its associations, we can mention the correlation with body mass index (BMI), clinical severity scores, and pulmonary function.^10^ ^,^ ^16^ ^,^ ^29^ Although the studies included in this review have used different ergometers (treadmill and cycle ergometer), both adopted incremental loading protocols, either by speed/slope or load (power).^11^ ^,^ ^22^ ^,^ ^23^ ^,^ ^24^ These protocols are the most recommended to measure physical fitness, considering that they lead the subjects to progressive cardiovascular stress, nearly exhausting them.^16^

All studies assessed pulmonary function in children and adolescents with PIBO, investigating the FEV_1_, FVC, and FEV_1_/FVC ratio. The typical ventilatory pattern of the disease is a severe ventilatory obstruction, which often does not respond to the treatments administered. The patient usually presents wheezing, tachypnea, dyspnea, and persistent cough for weeks or months after the initial infection. After the initial attack, the disease can persist for years, with exacerbations by viral infections, resulting in atelectasis and pneumonia.^30^ The present study identified a moderate/severe obstructive pattern, based on spirometry results, which corroborates the description of the disease and other previous studies.^31^ ^,^ ^32^ ^,^ ^33^ An important fact found in patients with chronic obstructive pulmonary disease^34^ was that 30% of the variability in exercise performance was attributed to airway obstruction observed with FEV_1_.^11^ ^,^ ^35^ These findings lead to the importance of the multi-professional team in thoroughly evaluating these subjects, taking into account that other factors, such as the usual level of physical activity, physical conditioning, and muscle capacity (peripheral and respiratory), can also have an important role in their aerobic fitness.

The studies included in this review seem to have some limitations, such as small sample size, variation in age group (5 to 23 years), lack of standardization of 6MWT results,^23^ and only one study having a control group.^22^ However, we believe that such restrictions did not influence the research question investigated due to the high methodological quality reached by the studies^11^ ^,^ ^22^ ^,^ ^23^ ^,^ ^24^ and to most of them presenting outcomes of exercise capacity after standardization of data by reference equations.^11^ ^,^ ^22^ ^,^ ^24^

In sum, the findings of this review showed reduced exercise capacity in children and adolescents with PIBO in most of the studies included. These results demonstrated the impact of this disease on the pediatric age group, considering that poor results in exercise tolerance are associated with worse outcomes regarding hospitalizations, the use of antibiotic therapy, and the reduced survival time of patients with respiratory diseases.^9^ ^,^ ^21^ ^,^ ^28^ Such fact should alert the professionals involved in this type of care, aiming to include them in pulmonary rehabilitation programs to decrease the impact of this disease, and improve the quality of life of these patients.
